# NO Synthesis in Immune-Challenged Locust Hemocytes and Potential Signaling to the CNS

**DOI:** 10.3390/insects12100951

**Published:** 2021-10-18

**Authors:** Stella Bergmann, Jan-Phillipp Gerhards, Anne Schmitz, Stefanie C. Becker, Michael Stern

**Affiliations:** 1 Institute for Physiology and Cell Biology, University of Veterinary Medicine Hannover, 30173 Hannover, Germany; stella.bergmann@tiho-hannover.de (S.B.); jan-phillipp97@web.de (J.-P.G.); as_anne.schmitz@web.de (A.S.); 2 Institute for Parasitology, University of Veterinary Medicine Hannover, 30559 Hannover, Germany; stefanie.becker@tiho-hannover.de

**Keywords:** nitric oxide, cGMP, hemocytes, insects, innate immunity, *Locusta migratoria*

## Abstract

**Simple Summary:**

Insects, in the same way as vertebrates, are exposed to a broad variety of pathogens but lack their adaptive immune system. Relying on their innate immune system, they respond to pathogens by phagocytosis, melanization, and the synthesis of antimicrobial or cytotoxic compounds. In this study, we evaluated the production of the cytotoxic gaseous radical nitric oxide (NO) in hemocytes, the immune cells of the model insect *Locusta migratoria* in response to various immune stimuli. Both sessile and circulating hemocytes responded to gram-negative *Escherichia coli* and gram-positive *Streptococcus suis* injection with a strong increase in NO production. In contrast, the gram-positive bacterium *Staphylococcus aureus* elicited only a minor response. In addition, bacteria were encapsulated by hemocytes. Since NO is an important neurotransmitter, NO-producing hemocytes were tested on the locust central nervous system (CNS) in an embryo culture model. CNS neurons responded with a distinct increase in production of the second messenger, cGMP. This is indicative of the influence of the immune response on the CNS. Our findings show that NO production in hemocytes and capsule formation need complex stimuli and contribute to the understanding of neuroimmune interactions in insects.

**Abstract:**

Similar to vertebrates, insects are exposed to a broad variety of pathogens. The innate insect immune system provides several response mechanisms such as phagocytosis, melanization, and the synthesis of antimicrobial or cytotoxic compounds. The cytotoxic nitric oxide (NO), which is also a neurotransmitter, is involved in the response to bacterial infections in various insects but has rarely been shown to be actually produced in hemocytes. We quantified the NO production in hemocytes of *Locusta migratoria* challenged with diverse immune stimuli by immunolabeling the by-product of NO synthesis, citrulline. Whereas in untreated adult locusts less than 5% of circulating hemocytes were citrulline-positive, the proportion rose to over 40% after 24 hours post injection of heat-inactivated bacteria. Hemocytes surrounded and melanized bacteria in locust nymphs by forming capsules. Such sessile hemocytes also produced NO. As in other insect species, activated hemocytes were found dorsally, close to the heart. In addition, we frequently observed citrulline-positive hemocytes and capsules near the ventral nerve cord. Neurites in the CNS of sterile locust embryos responded with elevation of the second messenger cGMP after contact with purified adult NO-producing hemocytes as revealed by immunofluorescence. We suggest that hemocytes can mediate a response in the CNS of an infected animal via the NO/cGMP signaling pathway.

## 1. Introduction

The broad variety of pathogens in the environment such as viruses, bacteria, and fungi affect vertebrates as well as invertebrates. In contrast to vertebrates, insects have an open circulatory system. The hemolymph dispenses hemocytes and potential pathogens throughout the body cavity [1,2,3,4].

Similar to all invertebrates, insects lack the adaptive immune system and rely solely on innate immunity [5]. The innate immune system can recognize and differentiate conserved motifs from cell wall components of bacteria such as lipopolysaccharides or peptidoglycans (PGN) or glucan from fungal cell walls [6,7]. Thereby, different pathogens elicit diverse immune responses mainly based on PGN, the amino acid sequence of which differs between gram-positive and -negative bacteria [8,9]. The main signaling pathways used in the immune system of insects driving antibacterial peptide gene expression are the Toll and IMD signaling pathways [10]. The Toll and IMD pathways are induced by gram-positive bacteria and fungi [11,12]. In contrast, gram-negative bacteria activate the IMD pathway [13,14,15]. In response to some bacteria, there is an overlap between the activation of IMD and Toll signaling [8,14,16,17].

The immune system of insects is divided into its cellular and humoral components [18,19]. The cellular immune response is mediated by hemocytes [20], which circulate freely or attach to tissues. Sessile hemocytes are found mainly on the abdominal wall, the trachea, and in regions around the heart in mosquitoes [21]. Sessile hemocytes can become circulating and vice versa [22].

The cellular defense strategies are phagocytosis, nodulation, and encapsulation [23]. Nodulation and encapsulation are often jointly termed capsule formation, a term which we will use throughout the text, and aim to isolate the invaders [24,25]. While phagocytosis is effective against smaller numbers of pathogenic particles, capsule formation is employed for larger numbers or bigger targets [24,26]. Infections in locusts induce capsule formation and melanization [27,28]. Additionally, distinct patterns of sessile hemocytes and capsules along the abdomen in locusts and various insect species have been reported [4,29].

As part of the humoral immune response, the gaseous radical nitric oxide (NO) is an important effector molecule (Nappi et al., 2000). It is synthesized by oxidation of L-arginine through the enzyme NO synthase (NOS) and NADPH as cofactor [30]. The amino acid L-citrulline is produced in equimolar amounts as a by-product [31]. Hemocytes, as well as fat body and midgut cells, were shown to be the source of NO during an acute infection. The synthetic activity of the induced NOS has been shown as Ca^2+^-dependent [30,32,33,34]. Elevated NO levels due to infection or immune stimulants were detected by the Griess reaction, for instance in *Anopheles gambiae*, *Anopheles stephensi*, *Drosophila melanogaster*, the flesh fly *Sarcophaga argyrostoma* or the moths *Mythimna separata* and *Galleria mellonella* [33,35,36,37,38,39,40,41]. Elevated NOS expression was shown by NADPH diaphorase activity [32,40,42], NOS-antibodies [33,42,43], or NOS mRNA levels [37,44,45]). Antibodies against the by-product of NO synthesis, L-citrulline, were, until now, only used in rats and the insect nervous system [31,46,47].

Due to its gaseous properties, NO can easily diffuse through membranes and is, therefore, a very efficient signaling molecule [30]. As a neurotransmitter, NO modulates information processing in the olfactory and visual systems, affects vesicle release at the neuromuscular junction, controls neurogenesis in the mushroom body, and plays a role in learning and memory formation [48,49,50,51,52,53,54]. The NO/cGMP pathway is also involved in mechanosensory information processing in the thoracic ganglia [55,56,57]. 

Since numerous arthropod species are carriers of various zoonotic diseases and spreading of the pathogen depends on the vector’s competence to eliminate the pathogen [58], the investigation of the insect immune response and altered signaling in the nervous system or even behavior is of great importance. In *Anopheles*, the synthesis of NO inhibits the development of the malaria pathogen [37]. In addition, behavioral changes could contribute to pathogen spreading. However, infections with viruses can also either increase or decrease feeding behavior and locomotor activity, or affect olfactory learning [59,60,61,62,63,64]. In addition, such effects in *Anopheles stephensi* for heat-inactivated *E. coli* were shown [65].

The specific mechanisms for these effects are often elusive. The effects of infections on the hemocyte-mediated immune response and the NO production as a possible mediator between the immune and nervous system are therefore of considerable interest. 

Locusts are among the most dangerous agricultural pests [66] and have proven to be useful model systems for research in neuronal function and development, including the role of NO [67] and for studying innate immunity [25,29,68,69,70,71,72]. Most of these studies focused on entomopathogenic fungi or soluble immune stimulants but rarely on bacteria. The present study aims to extend these studies on bacteria-induced immune responses, which have up to now mainly been investigated in dipterans and lepidopterans. We attempt to precisely localize the source of NO production and quantify it on a cellular level, using citrulline immunofluorescence, and aim to elucidate a potential NO/cGMP-based mechanism of immune system to CNS signaling during an infection. Here, we demonstrate a strongly elevated and quantifiable NO synthesis in circulating hemocytes as well as in sessile hemocytes of *L. migratoria* stimulated by the injection of heat-inactivated bacteria. We found NO-producing sessile hemocytes not only near the dorsal vessel and the hematopoietic tissue but also on the ventral diaphragm and attached to the central nervous system. We demonstrate the elevation of the second messenger cGMP in CNS neurons and their processes in locust embryos treated with NO-synthesizing-activated hemocytes.

## 2. Materials and Methods

### 2.1. Rearing of Locusta Migratoria

Experiments were conducted on first instar and adult female and male *Locusta migratoria*. First instar locusts were used, preferably a few hours after eclosion and after they turned to a dark color but not older than 48 h post eclosion, to minimize any possible natural infection that could interfere with the experiments. Adult locusts were chosen from three days to two weeks after final molt took place. Locusts were reared under a 12 h light/12 h dark cycle at 30 °C in crowded culture. Before dissection, animals were cold anesthetized on ice. Each experiment was repeated independently at least three times, for first instars on batches of sibling animals hatched from the same egg pod divided into different experimental and control groups. For quantification, data from all experiments of the same type were pooled. Exact numbers of preparations for each experiment are given in the supplementary information, Tables S2–S4. All chemicals were bought from Sigma, if not stated otherwise.

### 2.2. Bacterial Growth and Infection

*Escherichia coli* (DH-α) were taken from a laboratory culture at the university’s Institute of Parasitology; *Streptococcus suis* (WT10) and *Staphylococcus aureus* were kind gifts by Peter Valentin-Weigand, Institute of Microbiology of our university. Bacteria cultivated in Luria Bertani’s broth (LB) for 18 h at 37 °C were heat-inactivated at 96 °C for 5 min. Proper inactivation was controlled by plating the inactivated bacteria on LB agar and checking for bacterial growth after 18 h at 37 °C. To calculate bacterial numbers, serial dilutions of living overnight culture were plated on LB agar and incubated overnight at 37 °C. The colony forming unites (CFU) were determined. 

Adult locusts were cold anesthetized and injected equivalents of 4.6 × 10^6^ CFU in 5 µL of adjusted inactivated bacterial suspension intrathoracically with a Hamilton syringe. Three individuals per treatment group were injected. First instar locusts were anesthetized with CO_2_ and injected with equivalents of 5 × 10^5^ CFU in 193.2 nL of adjusted inactivated bacterial suspension intrathoracically with a Nanoject II (Drummond) using fine glass capillaries. Controls were sham injected with either LB or locust saline (150 mM NaCl, 3.1 mM KCl, 1 mM MgCl_2_, 5.4 mM CaCl_2_, 2 mM NaOH, 5 mM TES, 5 mM glucose, 100 mM sucrose, pH 7.2) or were left uninjured (naive). Treated locusts were kept at room temperature with fresh wheat grass ad libitum for 6 h (only first instar) or 24 h.

### 2.3. Inhibition of Nitric Oxide Synthase Activity

First instar locusts were injected with either 28 µg of D-NG-monomethyl arginine acetate (D-NMMA, Biomol) or of L-NG-monomethyl arginine acetate (L-NMMA, Biomol) solved in locust saline to obtain a concentration of 5 mM of inhibitor or inactive equivalent in the locust together with 5 × 10^5^ CFU of heat-inactivated *E. coli* in an injection volume of 193.2 nL with the Nanoject II. Locusts were dissected after 6 and 24 h. 

### 2.4. Injection of Diverse Immune Stumuli

For testing different immune stimuli or pathogenic agents, lipopolysaccharide (LPS), muramyl dipeptide (MDP), polymethyl methacrylate (PMMA) beads, and Rift Valley Fever Virus (RVFV) particles were used. First instar locusts were anesthetized with CO_2_ and injected with 3.75 × 10^5^ focus forming units (FFU) of RVFV suspension from a C6/36 cell line in 1 µL for anti-citrulline staining after 6 and 24 h or with 2.9 × 10^5^ FFU RVFV suspension from a BHK cell line in 1 µL for anti-RVFV staining after 1 h intrathoracically with a Nanoject II (Drummond). Controls were sham injected with the corresponding cell culture supernatant.

Solutions of LPS (516 µg/mL or 51.6 µg/mL, lipopolysaccharide from *Escherichia coli* O111:B4), MDP (580 µg/mL, N-acetyl-L-alanyl-D-isoglutamine hydrate) and 4.6 × 10^5^ 2 µm or a maximum of 460 20 µm fluorescent beads (unmodified PMMA beads PolyAn Blue, PolyAn GmbH, Berlin, Germany) were injected in a volume of 193.2 nL in first instar locusts. MDP solution and 4.1 × 10^6^ fluorescent PMMA beads were injected in a volume of 5 µL in adult locusts. Controls for first instar or adult locusts were injected with 193.2 nL of the respective solvent (sham) or were left uninjured. First instar locusts were dissected after 6 and 24 h and adult locusts after 24 h.

### 2.5. Dissection of First Instar Abdomen

First instar locusts were cold anesthetized and dissected in cold phosphate buffered saline (PBS, pH 7.4). Head and last abdominal segment including gut were removed. Abdomens were separated from thorax and cut along the lateral line. Abdomens were clamped to a Sylgard dish with minutian pins. Larger tracheae were removed. The abdomens were fixed with 4% paraformaldehyde for RVFV immunostaining or with 4% paraformaldehyde with 0.0125% glutaraldehyde in PBS for citrulline immunostaining for 30 min and for 15 min with PBS containing 10% methanol and 10% of a 37% formaldehyde solution (Roth), for NADPH diaphorase (NADPHd) activity staining.

### 2.6. NADPH Diaphorase Activity Staining

Fixated abdomens were washed twice with Tris buffer 0.1 M with 0.1% Triton X-100 (pH 7.8) for 15 min. Staining was performed with a solution of 2 mg β-nicotinamide adenine dinucleotide 2′-phosphate (NADPH) and 2 mg nitrotetrazolium blue in 10 mL of Tris buffer in darkness at room temperature until, after approximately one hour, a blue staining was visible. Abdomens were washed with distilled water four times for 15 min. Clearing was conducted with methanol/acetic acid (1:3) for three minutes and, subsequently, with methanol three times for three minutes. Methanol was exchanged for cedar oil until remaining methanol was evaporated. Abdomens were coverslipped with cedar oil and examined under bright field (Axioskop, Carl Zeiss Microscopy, Oberkochen, Germany).

### 2.7. RVFV Immunofluorescence

Fixated abdomens were permeabilized with PBS with 0.1% Triton X-100 (PBS-T) and 0.3% saponin for 30 min. Washing was performed with PBS-T on abdomens for 10 min. Blocking solution (PBS-T with 5% normal goat serum, Invitrogen, Thermo Fisher, Waltham, MA, USA) was applied for 30 min. Mouse-anti-RVFV antibody Gn164b [73], which stains RVFV glycoprotein in various tissues of different mammalian and insect species [74], was used 1:50 in blocking solution on abdomens overnight. Subsequently, abdomens were washed twice with PBS-T for 5 min. AlexaFluor568 goat anti-mouse IgG (Invitrogen) was used as secondary antibody (1:333) with DAPI (0.1 µg/mL) and Streptavidin AlexaFluor488 conjugate (Invitrogen) (1:200) in blocking solution overnight. Abdomens were washed with PBS-T for 5 min and rinsed with distilled water. Preparations were cleared in 50% glycerol for 30 min and then coverslipped with 90% glycerol.

### 2.8. Extraction of Adult Circulating Hemocytes

Generation of hemocyte primary culture was conducted according to a modified protocol from Huxham and Lackie [75]. Cold anesthetized adult locusts were surface sterilized with 70% ethanol and injected with 1 mL of ice-cold sterile anticoagulant (26 mM citric acid, 30 mM sodium citrate, 60 mM sodium chloride, 100 mM glucose, 10 mM EDTA, pH 4.6) into the thorax. The hind leg was cut and hemolymph was collected in a 2.0 mL reaction tube. Hemolymph was centrifuged at 250 g for 5 min. The supernatant was discarded. The hemocytes of a treatment group were pooled by resuspending the pellets successively in the same 500 µL of cold anticoagulant. The pooled cells were centrifuged for 5 min at 250 g and were washed a second time. The pellet was finally resuspended in 1 mL of cold Schneider’s Insect Medium (Invitrogen). The cell number was adjusted and hemocytes were immediately plated at 100,000 cells in 500 µL Schneider’s Insect Medium per well on four-well slides (Sarstedt). For every treatment group of a replicate, all wells of a 4-well slide were seeded. Hemocytes were left to adhere for one hour at room temperature and subsequently fixed with 4% paraformaldehyde with 0.0125% glutaraldehyde for 10 min. On average, 337,659 ± 20,252 hemocytes/locust were recovered per experiment throughout the study with no statistical differences between treatment groups (supplemental information Figure S2). In one experiment, hemocytes were collected of first instar locusts injected with 4.6 × 10^5^ fluorescent beads (2 μm unmodified PMMA beads PolyAn Blue, PolyAn GmbH, Berlin, Germany) 24 h post injection (hpi). After injection with cold anticoagulant, the middle leg was cut, hemolymph was obtained from the thoracic opening and collected in a drop of Schneider’s medium.

### 2.9. Citrulline Immuofluorescence

For citrulline immunolabeling, abdomens were permeabilized with PBS with 0.1% Triton X-100 (PBS-T) and 0.3% saponin for 30 min. Washing was performed with PBS-T on abdomens for 10 min and on hemocyte primary culture for 5 min. Blocking solution (PBS-T with 5% normal goat serum, Invitrogen) was applied for 30 min on abdomens and 5 min on hemocyte primary culture. Mouse-anti-citrulline antibody ([31], a kind gift by G.A. Holstein and G.P. Martinelli, New York, RRID:AB_2314197) was used 1:200 in blocking solution on abdomens overnight and on hemocyte primary culture for 30 min. Subsequently, both preparations were washed twice with PBS-T for 5 min. AlexaFluor568 goat anti-mouse IgG (Invitrogen) was used as secondary antibody (1:333) with DAPI (0.1µg/mL) or Ethidium D (1:000) and either Streptavidin AlexaFluor488 conjugate (Invitrogen) (1:200) or Phalloidin-iFluor 488 Reagent (Abcam) (1:200–500) in blocking solution for a minimum of 4 h on abdomens and for 30 min on hemocyte primary culture. Again, preparations were both washed with PBS-T for 5 min and rinsed with distilled water. Hemocyte primary culture was coverslipped with 90% glycerol. Abdomens were cleared in 50% glycerol for 15 to 30 min and then coverslipped with 90% glycerol.

### 2.10. Evaluation of Citrulline-Positive Hemocytes and Abdomens

Images were acquired with an Axiocam 506 color camera linked to fluorescence microscope Axioskop and ZEN 2012 blue edition software (Carl Zeiss Microscopy). Images of hemocyte culture were taken at equal excitation intensity and exposure settings to acquire comparable images for counting citrulline-positive cells with a standardized threshold in ImageJ 1.52a (Wayne Rasband, National Institutes of Health). In order to determine the proportion of citrulline-positive hemocytes in the locust primary culture, either three (with four wells per treatment group) or ten image sections (with two wells per treatment group) per well were selected at random only at the basis of present DAPI positive cells. The number of DAPI and citrulline-positive hemocytes in the respective sections were counted automatically in ImageJ using an adjusted and fixed intensity threshold. Percentage was calculated per image by dividing number of citrulline-positive hemocytes by the number of DAPI positive hemocytes. Averages of 3 to 6 independent experiments were plotted and tested for significant differences between treatment groups using a Kruskal–Wallis with Dunn’s multiple comparison test in GraphPad Prism 9.

Images of citrulline immunofluorescence of first instar abdomens were also taken at equal excitation intensity and exposure settings. Dorsal and ventral parts of abdomens were checked for capsules indicated by surrounding hemocytes and by their brown core in the green channel and likewise for at least three citrulline-positive hemocytes indicated by their polymorph shape and a bright cytosolic anti-citrulline immunofluorescence. Images of D-NMMA- and L-NMMA-treated first instar abdomens were randomized and double blind checked by an additional person. Results are represented as percentage of dorsal or ventral abdomens with at least three citrulline-positive hemocytes or with noticeable capsules of all abdomens in a treatment group. To test for significant difference between the groups and time points, Fisher’s exact test was used (GraphPad Prism 9).

### 2.11. Pre-Adsorption Control for Anti-Citrulline Antibody

Pre-adsorption control was performed after a modified protocol from Hoskins et al. (1986) [76]. Either arginine or citrulline were weighed to an equivalent of 100 µM and coupled to 12 mg bovine serum albumin (BSA) in 2 mL PBS. In aliquots of 5 µL, a total of 40 µL of 25% glutaraldehyde was added while stirring. After 30 min, 6 mL PBS was added and the solution was allowed to stand for another 30 min to facilitate the coupling at room temperature. For inactivation of the remaining glutaraldehyde, 1.6 mL of 1 M glycine solution was added for one hour at room temperature. Samples were dialyzed against PBS overnight and, subsequently, twice against fresh PBS for two hours each. 

Citrulline antibody solution was preincubated with conjugated amino acid solution (1:200) and tested on dissected abdomens of 5 × 10^5^ CFU heat-inactivated *E. coli*-injected first instar locusts according to the staining regime described above. Preincubation with conjugated citrulline resulted in no anti-citrulline stained hemocytes while capsules were clearly visible, whereas preincubation with conjugated arginine showed a strong anti-citrulline immunofluorescence similar to abdomens stained with untreated citrulline antibody solution (see supplemental information, Figure S1). This indicates a strong specificity of the employed anti-citrulline antibody on locust abdominal tissue.

### 2.12. Embryo Dissection

Fillet preparations of locust embryos staged to ca. 63.5% of completed embryogenesis according to Bentley et al. (1979) [77] were prepared according to Stern and Bicker (2008) [78]. In brief, legs and head were removed, the body wall was cut open next to the dorsal heart tube and the gut was removed. The preparations were then stuck to a slide with liquid Sylgard 184 (Farnell, Poing, Germany) in a Sylgard cavity. In total, 150 µL Schneider’s Insect medium was added and the preparations were spread on the slide between Sylgard and medium. The ventral diaphragm was carefully removed with fine forceps to expose the CNS.

### 2.13. cGMP Immuostaining and Assessment

Before fixation, embryo preparations were incubated with 1 mM 3-isobutyl-1-methylxanthine (IBMX) in Schneider’s Insect medium for 30 min to inhibit phosphodiesterases. Hemocytes from naive or *E. coli*-treated adult locusts were collected as described above, and finally resuspended in Schneider’s Insect medium with 1 mM IBMX. A total of 150 µL hemocyte solution was applied for 30 min. As a positive control, the NO donor sodium nitroprusside (SNP, 100 µM) was added instead of hemocytes. Preparations were then fixated with 4% PFA with 0.0125% glutaraldehyde for 45 min. Permeabilization was performed by PBS-T with 0.3% saponin and subsequently washed with PBS-T for 5 min twice. Preparations were blocked with PBS-T with 5% normal horse serum (Vector Laboratories, Burlingame, CA, USA.). The primary antibodies sheep-anti-cGMP (1:20,000) (a kind gift by J. de Vente, RRID:AB_2314152) and mouse-anti-citrulline (1:200) in PBS-T with 5% normal horse serum were incubated overnight. Embryos were then washed four times for 15 min with PBS-T. The secondary antibody donkey-anti-sheep AlexaFluor488 (Invitrogen) (1:333) was applied for 2 h. Preparations were then washed again four times with PBS-T. The next secondary antibody goat-anti-mouse (Invitrogen) (1:333) was incubated for 2 h. After this, embryos were washed twice with PBS-T for 10 min and with PBS for 10 min. Clearing was obtained with 50% glycerol in PBS and preparations were coverslipped in 90% glycerol in PBS. Pictures were taken with an Axiocam 506 color camera linked to fluorescence microscope Axioskop and ZEN 2012 blue edition software (Carl Zeiss Microscopy). The eight connectives between abdominal ganglia 3 and 7 of each embryo were examined for presence of cGMP-positive neurites. To test for significant difference between the groups, Fisher’s exact test was used (GraphPad Prism 9).

## 3. Results

Our findings are based on observations on a total of 926 first instar abdomens, 168 adult floating hemocyte cell cultures, 1004 images of hemocyte cell cultures, and 27 embryo fillet preparations. Exact numbers are given in Tables S1–S4, online supplementary information.

### 3.1. Abdomens of First Instar Locusts

**Figure 1:**
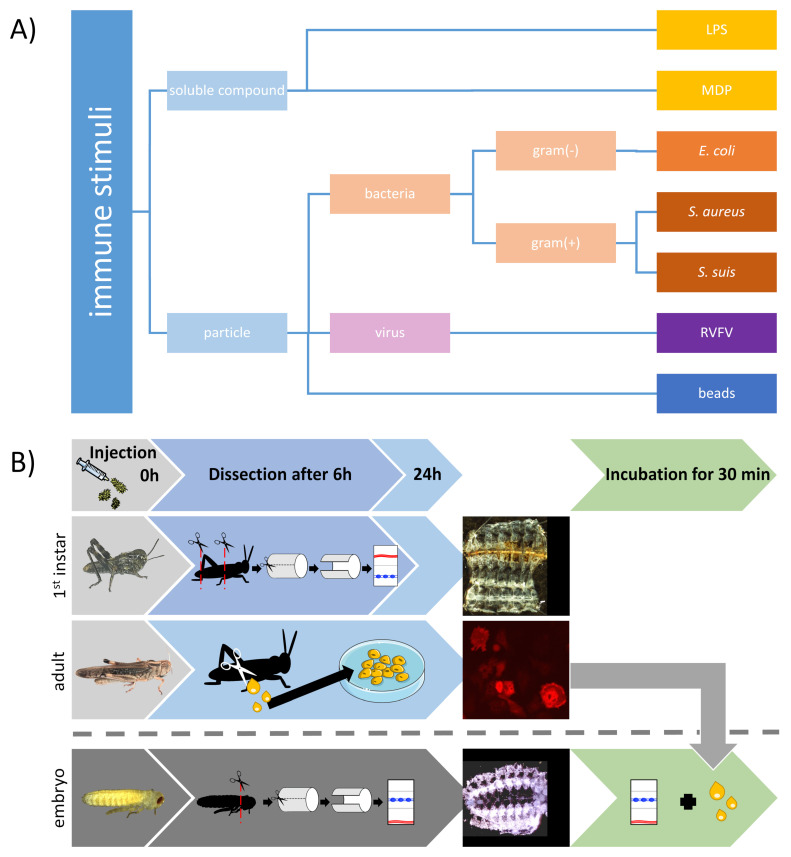
Employed immune stimuli and experimental schedule. (**A**) Immune response of locusts towards diverse soluble or particulate stimuli with different conserved motifs, e.g., in gram-positive and gram-negative bacteria or without any organic traits represented by the PMMA beads were tested. (**B**) Three different stages of *Locusta migratoria* were used. For investigation of the sessile hemocytes, first instar larval locusts were injected with a stimulant and dissected after 6 and 24 h to expose the dorsal and ventral diaphragm with the dorsal vessel (red) and the CNS (blue) for later immunolabeling. Circulating hemocytes were obtained by cutting the adult locust’s hindlegs 24 h post injection (hpi) and collecting the hemolymph. Hemocyte primary culture was established for later immunolabeling. The effect of adult stimulated hemocytes on the insects CNS was studied on dissections of locust embryos with exposed nervous system (blue).

**Figure 2:**
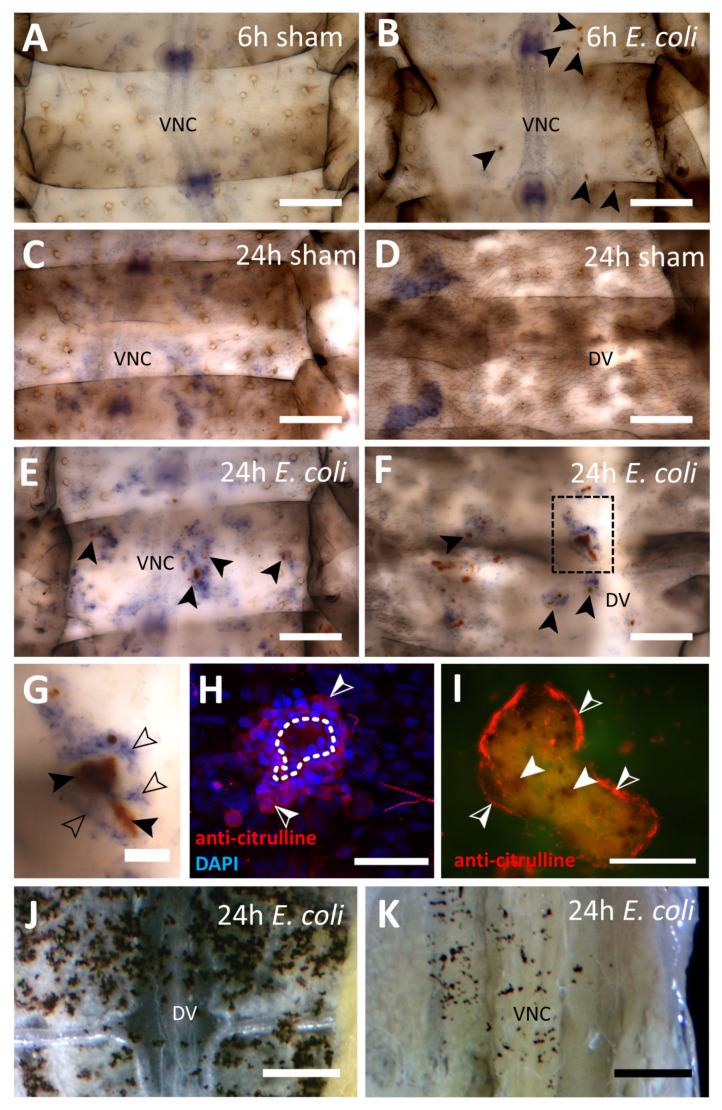
Capsule formation in *L. migratoria* abdomens (**A**–**F**) NADPH diaphorase staining of first instar locust abdomens either sham injected or injected with heat-inactivated *E. coli*; filled arrows point to melanized capsules; scale bar: 200 µm (**G**) enlargement of the indicated area in (**F**), filled arrows point to melanized mass, empty arrows point to NADPHd positive hemocytes; scale bar: 50 µm (**H**) immunofluorescence image of citrulline-positive hemocyte surrounded capsule (indicated by dotted line) in multiple layers in a first instar abdomen, half-filled arrows point to citrulline-positive hemocytes; scale bar: 50 µm (**I**) immunofluorescence image (from a different preparation than in (**H**)) of large encapsulated, partially melanized bacterial mass (observable in green background fluorescence) surrounded by citrulline-positive hemocytes, filled arrows point to melanized spots, half-filled arrows point to citrulline-positive hemocytes; scale bar: 200 µm (**J**,**K**) melanized capsules in the abdomens of adult locusts, (**J**) dorsal, (**K**) ventral; scale bar: 1mm. Abbreviations: DV = dorsal vessel; VNC = ventral nerve cord.

In order to examine the function of the immune response in the grasshopper *Locusta migratoria*, we tested various immune stimuli (Figure 1A). Following an injection of heat-inactivated *E. coli*, the formation of melanized capsules enclosing bacterial aggregates were observed in the first instar nymphs already after the first chosen time point of 6 h (Figure 1B and Figure 2B). The melanized capsules were still present 24 hpi (Figure 2E–G). Capsules were found on both the dorsal side of the abdomen around the dorsal vessel (Figure 2F) and on the ventral side, frequently near the ventral nerve cord (Figure 2E). Injection of *E. coli* into adult locusts generated comparable results 24 hpi (Figure 2J,K). As reported before [47], NADPHd staining revealed the presence of NOS in the ventral nerve cord in all preparations (Figure 2A–C,E). In contrast to 6 hpi, capsules at 24 hpi were surrounded by NADPHd positive hemocytes, which indicates NOS presence (Figure 2G). Immunofluorescence not only showed in more detail multiple layers of hemocytes wrapped around the foreign mass to build a capsule but also the production of NO by citrulline immunofluorescence (Figure 2H,I). Sham injected control animals did not show capsule formation (Figure 2A,C,D). 

#### 3.1.1. Inhibition of NOS

**Figure 3:**
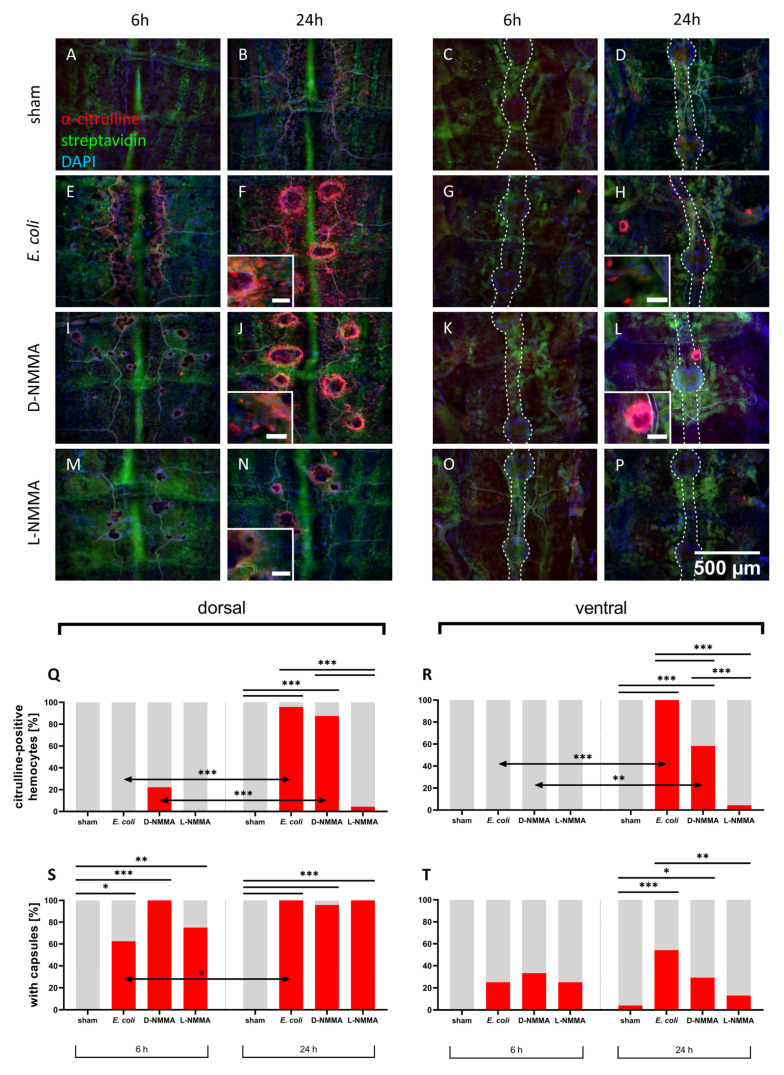
Citrulline-immunoreactivity and capsule formation on first instar locust body walls. (**A**–**P**) Fluorescent photomicrographs of (**A**–**D**) sham-injected (**E**–**H**) heat-inactivated *E. coli* injected (**I**–**L**) D-NMMA and heat-inactivated *E. coli* injected (**M**–**P**) L-NMMA and heat-inactivated *E. coli* injected preparations labeled for citrulline (red), DAPI (blue), and streptavidin (green) to label the dorsal vessel and fat body cells. Scale bar in the enlarged insets in (**F**,**H**,**J**,**L**): 50 µm, ventral nerve chord indicated with dotted line. (**Q**,**R**) percentage of dorsal and ventral abdomens with citrulline-positive hemocytes (**S**,**T**) percentage of dorsal and ventral abdomens with capsules, pooled data from three independent experiments, statistical test: Fisher’s exact test, ns > 0.05 (not indicated), *p* ≤ 0.05 (*), *p* ≤ 0.01 (**), *p* ≤ 0.001 (***).

To confirm that the citrulline detected by the antibody is indeed the by-product of NO synthesis by the enzyme NOS, we injected it together with the heat-inactivated *E. coli,* the NOS-inhibitor L-NMMA, or its inactive enantiomer D-NMMA. The abdomens of the first instar nymphs injected with *E. coli* served as positive controls and corresponded to the findings of the NADPHd staining. Capsules started to form 6 hpi with a percentage of 62.5% of capsule-containing dorsal abdomens and 25.0% on the ventral side (Figure 3E,S,T). The groups co-injected with L- or D-NMMA showed capsule formation as well (Figure 3I,M,S,T). Citrulline-positive hemocytes were rarely observed at this time point and no significant differences to the sham-injected control was detected (Figure 3E,I,M,G,K,O,Q,R). The percentage of dorsal abdomens with capsules 24 hpi increased in all bacteria-injected groups to reach between 95.8 to 100.0%, which was in the case of *E. coli*-injected animals a significant increase (Figure 3S). Nearly all abdomens of locusts injected with *E. coli* alone contained citrulline-immunoreactive hemocytes (Figure 3F,H,Q,R). For ventral abdomens, the inhibitor co-injected group showed significantly less capsule formation than animals injected with *E. coli* alone (Figure 3T). Occurrence of citrulline-positive hemocytes was rarely observed in dorsal and ventral parts of inhibitor co-injected locusts (4.3%) and no significant difference to the sham-injected controls was detected (Figure 3N,P,Q,R). On the other hand, the group co-injected with the inactive enantiomer, D-NMMA, showed significantly more abdomens with citrulline-positive hemocytes (Figure 3J,L,Q,R). Both D-NMMA- and *E. coli*-injected locusts frequently contained citrulline-positive hemocytes near or even on the ventral nerve cord (Figure 3H,L). Taken together, the NOS inhibitor group showed a decreased proportion of abdomens with citrulline-positive hemocytes but still capsule formation, whereas the D-NMMA group was mostly comparable to the *E. coli* injected animals. Furthermore, pre-adsorption controls could confirm specificity of the anti-citrulline antibody (Supplementary Figure S1).

#### 3.1.2. Bacterial Immune Challenge

**Figure 4:**
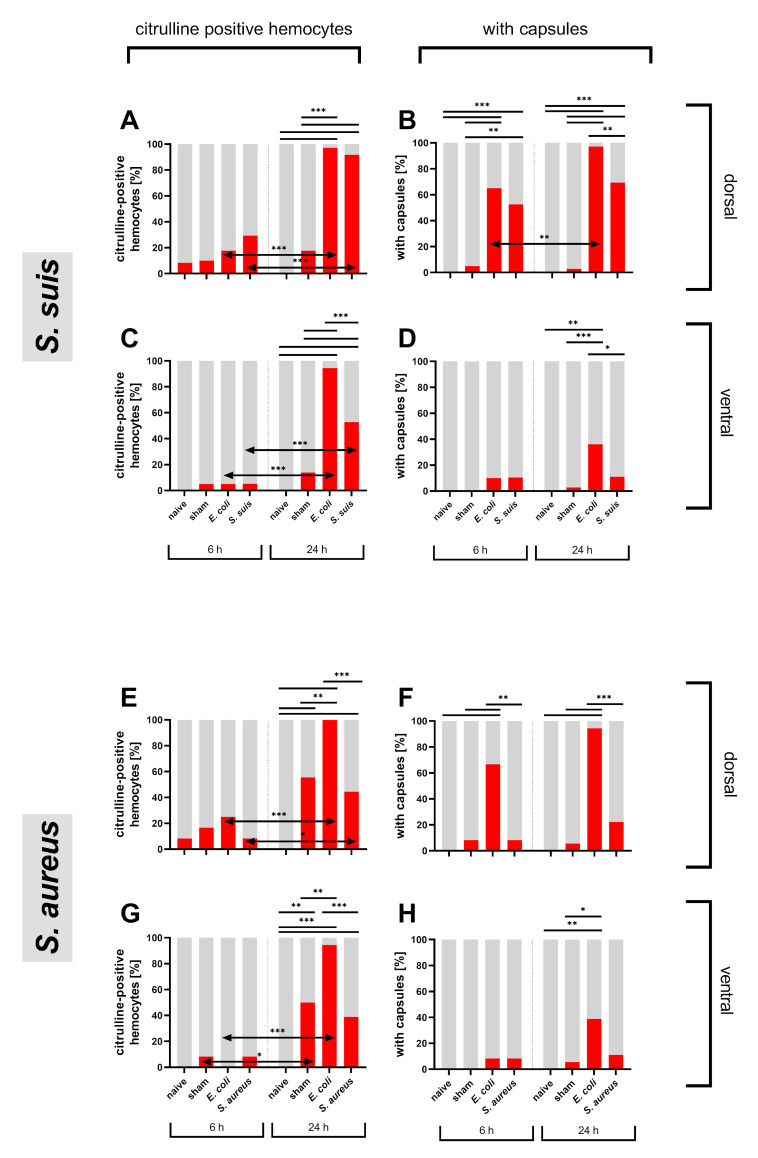
Injection of abdomens of first instar *L. migratoria* with heat-inactivated *E. coli*, *S. suis* and *S. aureus*. Naive (uninjected) animals were included to ensure absence of any natural infection. Shown is the percentage of dorsal and ventral abdomens with citrulline-positive hemocytes in (**A**,**C**) in the *S. suis* trial and in (**E**,**G**) in the *S. aureus* trial as well as the percentage of dorsal and ventral abdomens with capsules in (**B**,**D**) in the *S. suis* trial and in (**F**,**H**) in the *S. aureus*, statistical test: Fisher’s exact test, ns > 0.05 (not indicated), *p* ≤ 0.05 (*), *p* ≤ 0.01 (**), *p* ≤ 0.001 (***).

After verifying the dependence of anti-citrulline immunofluorescence on NO production in locusts injected with gram-negative *E. coli*, the gram-positive bacteria *S. suis* and *S. aureus* were tested in the abdomens of first instar nymphs and their effects compared to *E. coli*. At 6 hpi, only a low percentage of abdomens showed citrulline-positive hemocytes with no significant differences between tested groups (Figure 4). For the dorsal side, the percentage of abdomens with citrulline-positive hemocytes remained below 30% in all groups tested (Figure 4A,E). On the ventral side, the percentage was even lower with a maximum of 8.3% (Figure 4C,G). At 24 hpi, nearly all *E. coli*-injected locusts showed citrulline-positive hemocytes on dorsal or ventral abdomens in both test series (Figure 4A,C,E,G). For the *S. suis* group, the portion of dorsal positive abdomens was comparable to the *E. coli* group with 91.7% and, on the ventral side, it was more than half of the abdomens (Figure 4A,C). *S. aureus*-injected locusts had a smaller portion, 44.4% on dorsal and 38.9% on ventral abdomens (Figure 4E,G). From 6 hpi to 24 hpi the portion of positive abdomens increased significantly dorsally and ventrally in *E. coli* injected animals as well as in *S. suis* injected locusts (Figure 4A,C,E,G). *S. aureus* injected animals showed a less prominent increase (Figure 4E,G).

Capsule formation was already present at 6 hpi with the highest portion in *E. coli* injected animals with around 65% and a similar high portion in the *S. suis* group with 52.6% in dorsal abdomens (Figure 4B,F). Again, *S. aureus*-injected animals showed no strong reaction to the stimuli (Figure 4F). In all treatment groups, fewer capsules were found ventrally than dorsally (Figure 4D,H). At 24 hpi, the portions of abdomens with capsules increased. Although abdomens of *S. suis*-injected animals showed significantly fewer capsules than *E. coli*-injected animals, they still contained significantly more capsules than the sham-injected group on the dorsal side in contrast to *S. aureus*-injected locusts, where capsule formation did not increase (Figure 4B,D,F,H).

In general, *S. suis*-injected locusts displayed a reaction more comparable to the *E. coli* group and a significantly stronger response than sham-injected locusts, whereas the injection of *S. aureus* did not provoke a significantly different response to the sham injection.

#### 3.1.3. Diverse Immune Stimuli

Further, different kinds of stimuli were tested representing different aspects of bacteria properties. LPS and MDP are soluble components of bacteria surface structures known for their immune stimulating effects in various species. In contrast, biologically inert PMMA beads only provide the mechanical stimulus of a bacteria or capsule sized particle. As a non-bacterial immune challenge, a virus with a known immunostimulant effect in other insects, RVFV, was chosen [79,80,81].

**Figure 5:**
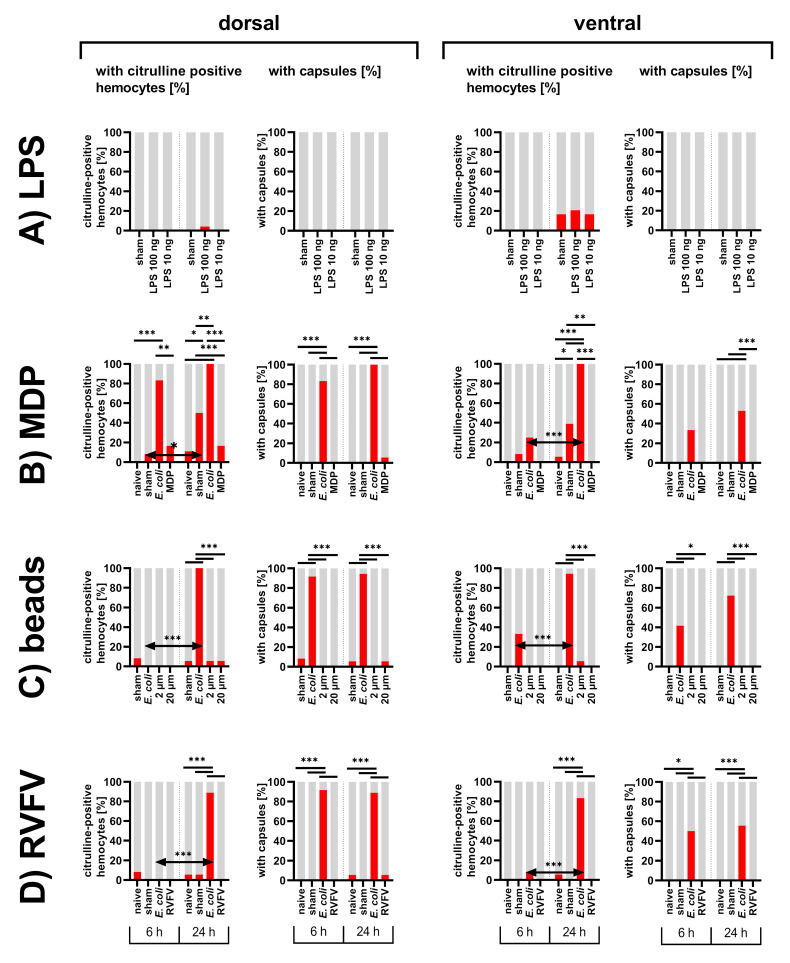
Abdomens of first instar *L. migratoria* injected with (**A**) LPS, (**B**) MDP, (**C**) PMMA beads, and (**D**) RVFV. Shown is the percentage of dorsal and ventral abdomens with citrulline-positive hemocytes and the percentage of dorsal and ventral abdomens with capsules, statistical test: Fisher’s exact test, ns > 0.05 (not indicated), *p* ≤ 0.05 (*), *p* ≤ 0.01 (**), *p* ≤ 0.001 (***).

Independent of the kind of immune stimulant, capsule formation in the treatment groups did not exceed that of the sham-injected group (Figure 5). Citrulline-positive hemocytes were also rarely observed in the treatment groups. Only in the sham controls for the MDP test set, did citrulline-immunoreactivity significantly increase, possibly due to contamination introduced by the injection (Figure 5B). Nevertheless, MDP-injected locusts displayed little citrulline-immunoreactivity and were more comparable to the naive animals (Figure 5B).

#### 3.1.4. Distribution of Particles in the Abdomen of First Instar Locusts

**Figure 6:**
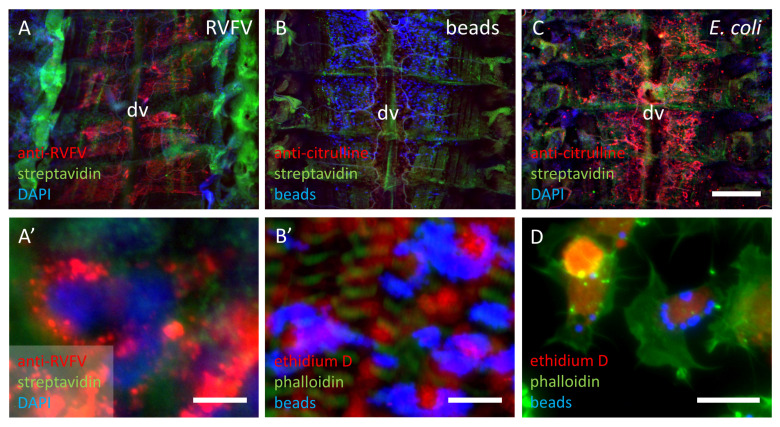
Distribution of injected particles in first instar locusts on dorsal abdomens and in hemocytes. (**A**,**A’**) abdomens of 2.9 × 10^5^ FFU RVFV-injected locusts 1 hpi (**A’**) scale bar: 10 µm (**B**,**B’**) abdomens of blue fluorescent 2 µm polymethyl methacrylate (PMMA) beads injected locusts 6 hpi, nuclei labeled in red with ethidium D (**B’**) scale bar: 20 µm (**C**) abdomen of 5 × 10^5^ CFU *E. coli*-injected locusts 24 hpi. (**D**) hemocytes with phagocytosed beads in primary cell culture of injected locusts 24 hpi, scale bar: 20 µm. dv = dorsal vessel, scale bar in (**C**): 500 µm, applies for (**A**–**C**).

Since injection of RVFV or PMMA beads did not elicit citrulline-immunoreactivity in hemocytes or capsule formation in abdomens of first instar nymphs, distribution of the injected particles was followed by fluorescence or immunofluorescence. *E. coli*-injected locusts showed most of the capsules and citrulline-positive hemocytes along the dorsal heart vessel, where the hematopoietic reticular cells reside (Figure 6C). This pattern is also reflected in the distribution of the 2 µm fluorescent beads and in the immunostaining of injected RVFV particles (Figure 6A,B). Both the fluorescent beads (6 hpi) and the RVFV particles (1 hpi) appear to be phagocytosed by the hematopoietic tissue (Figure 6A’,B’) as well as by the hemocytes (24 hpi) (Figure 6D).

### 3.2. Hemocyte Primary Culture of Adult Locusts

**Figure 7:**
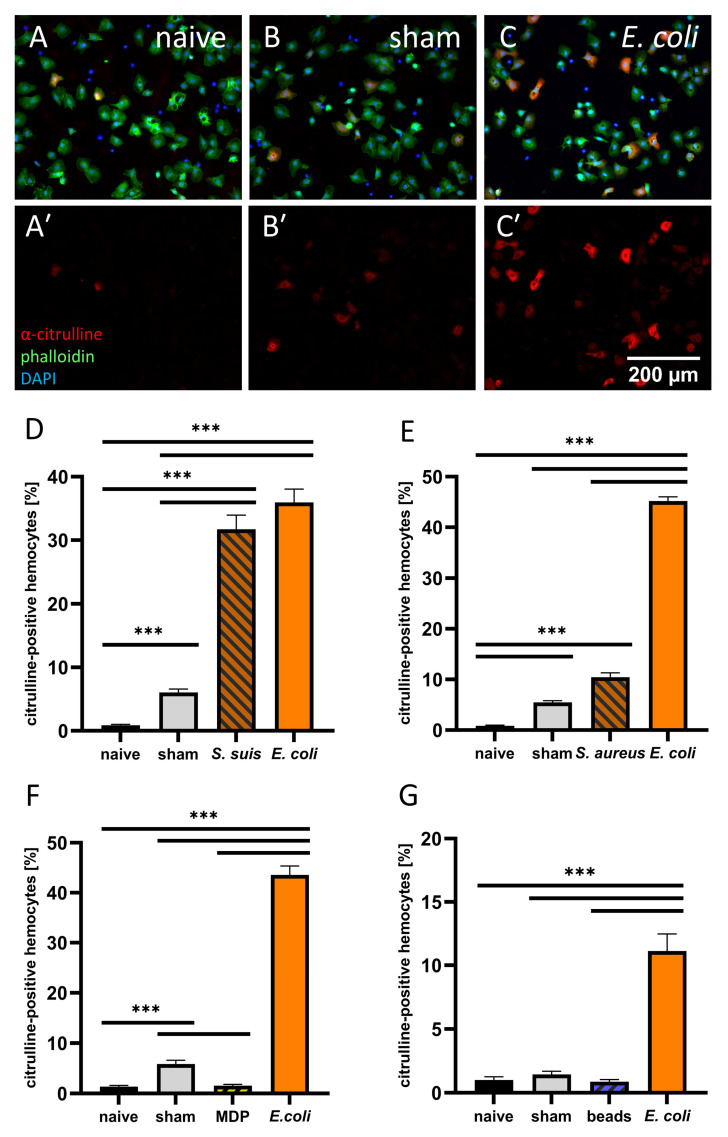
Hemocyte primary culture of adult *L. migratoria* 24 hpi. (**A**–**C**) composites of immunofluorescence images from a-citrulline, phalloidin, and DAPI staining of hemocytes of naive locusts and sham or heat-inactivated *E. coli*-injected locusts, (**A’**–**C’**) citrulline immunofluorescence alone of the same cultures as in (**A**–**G**), mean percentage (±SEM) of citrulline-positive hemocytes of *S. suis* (n = 94–97), *S. aureus* (n = 60), MDP (n = 60), or PMMA beads (n = 35–37) injected locusts from three to six biological replicates; statistical test: Kruskal–Wallis test with Dunn’s multiple comparison test *p* ≤ 0.001 (***).

NO synthesis in sessile hemocytes in the locust abdomens was successfully localized and evaluated by simple qualitative criteria. For a more quantitative analysis, we also tested circulating hemocytes of adult locusts (Figure 7). These primary cell cultures are applicable to an unbiased, automatic quantitative assessment by a defined threshold. As shown for sessile hemocytes, a large proportion of circulating hemocytes of adult locusts 24 hpi of heat-inactivated *E. coli* reliably displayed citrulline-immunoreactivity (44.18 ± 2.87% SEM, 10 independent experiments). Hemocytes from *E. coli*-injected locusts were, therefore, used as positive controls in all following experiments. *S. suis* elicited a strong NO synthesis in circulating hemocytes (31.71 ± 2.84% SEM (n = 94)) comparable to *E. coli* (35.96% ± 2.10% SEM (n = 94)) (Figure 7D). The gram-positive bacteria *S. aureus*, though, induced fewer hemocytes to synthesize NO (10.46 ± 0.87 SEM (n = 60)), but, in contrast to the response in sessile hemocytes, there was a significantly higher portion of citrulline-positive cells than in sham-injected locusts (Figure 7E). As for sessile hemocytes, the soluble bacteria component MDP did not promote NO synthesis (Figure 7F). Additionally, 2 µm PMMA beads did not induce NO synthesis in circulating hemocytes (Figure 7G) but were phagocytosed by the hemocytes (Figure 6D).

### 3.3. cGMP-Positive Axons in Locust Embryos

**Figure 8:**
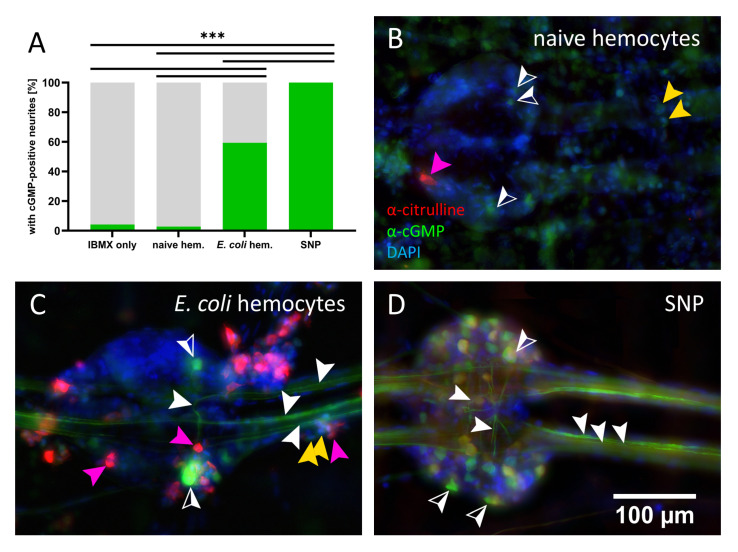
cGMP immunofluorescence of locust embryo fillet preparations. (**A**) percentage of connectives containing cGMP-positive neurites in embryos treated with of IBMX only (n = 24 connectives), IMBX + naive hemocytes (n = 72), IMBX + *E. coli* treated hemocytes (n = 96) and IMBX + SNP (100%, n = 24), from three biological replicates (**B**–**D**) ganglion and connectives with cGMP-positive cell bodies and neurites. White, half-filled arrow head: cGMP-positive cell bodies; white arrowhead: cGMP-positive neurite; pink arrowhead: citrulline-positive hemocyte; yellow arrowhead: citrulline-negative hemocyte; statistical test: Fisher’s exact test, ns > 0.05 (not indicated), *p* ≤ 0.001 (***).

The frequent observation of multiple citrulline-positive hemocytes or even hemocyte-coated capsules on the ventral side, often directly attached to the CNS, raised the question of a possible impact of NO released from hemocytes on the nervous tissue. We addressed this question in completely sterile locust embryos at a stage where none or very little NO synthesis and, thus, citrulline is present [47]. Locust embryos above 60% of development can be easily dissected as fillet preparations (Figure 1B) with the nervous system (five abdominal ganglia connected by a total of eight connective nerves) exposed and available for pharmacological intervention [78]. We treated embryo fillet preparations preincubated with the phosphodiesterase inhibitor, IBMX, for 30 min. with hemocytes of naive or *E. coli*-treated adult locusts. As a negative control, we incubated embryos with IBMX only, and as a positive reference we used the NO donor, SNP. Using an antibody against cGMP, accumulated cGMP could be detected in cell bodies as well as commissural and connective neurites (Figure 8). In both negative controls, IBMX only, and naive hemocytes treated embryos, only a small percentage of connectives (fewer than 5%) displayed cGMP-positive neurites (Figure 8A). Occasionally, a few cGMP-positive somata could be found as well as citrulline-positive hemocytes in preparations treated with naive adult hemocytes (Figure 8B). The NO donor SNP induced strong cGMP production in numerous cell bodies in the ganglia and in multiple neurites of a connective in all tested preparations (Figure 8A,D). In the group incubated with hemocytes from adult *E. coli*-injected locusts, citrulline-immunoreactive hemocytes were found all over the preparation and along the CNS (Figure 8C). In nearly 60% of connectives, they caused one or multiple neurites to accumulate cGMP, and strongly cGMP-positive somata were also observable (Figure 8A,C).

## 4. Discussion

### 4.1. Localization of Nitric Oxide Synthesis in Response to an Immune Stimulus

Nitric oxide is known to have multiple functions as a signaling molecule in the insect nervous system [82,83] and as an effector molecule in the immune system [3,43,51]. Here we studied NO production in insect hemocytes in response to an immune stimulation and the induction of cGMP synthesis in the central nervous system caused by NO producing hemocytes.

Since nitric oxide is a gaseous messenger, it is hard to localize in tissue. Antibodies against NOS protein or the NADPH diaphorase staining method localize potential NO producing cells, but since NOS activity in insect hemocytes is calcium-dependent [32], those techniques are not sufficient to quantify NO synthesis. As a consequence, NOS activity is often measured indirectly through quantification of the degradation product of NO, nitrite, employing the Griess reaction in hemolymph or tissue lysates [32,36,41]. This enables the detection of possible NO synthesis but not its cellular localization. Fluorescent NO indicators such as diaminofluoresceins [84] can principally be used to localize NO production on the cellular level in tissues, but have proven too insensitive to quantify pathogen-induced changes in NOS activity in insect hemocytes [85]. Immunolabeling of the by-product of NO synthesis, L-citrulline, indicates not only the presence and localization of NOS [31] but also its differential activity in the tissue. After crushing NOS expressing axons in locust embryos, they begin to accumulate citrulline within 30 min after the lesion, and this response is blocked by the inhibition of NOS [47]. In the present study, we have shown that after challenging first instar locusts with *E. coli* or *S. suis*, citrulline immunoreactivity was induced in both circulating and sessile hemocytes within 24 h. The inhibition of citrulline induction by the NOS-inhibitor L-NMMA, but not by its inactive enantiomer D-NMMA (Figure 3), strongly supports the notion that citrulline accumulation actually reports pathogen-induced NOS activity in locust hemocytes. Even though the integration time of the citrulline signal is not exactly known, it became clear that no substantial NO synthesis was detected six hours after the immune challenge, whereas many hemocytes displayed citrulline-immunoreactivity after 24 h (Figure 4). Since capsule formation already commenced six hours post injection, when no citrulline immunoreactivity was observed yet, NO is unlikely to be a prerequisite for pathogen encapsulation.

A similar time course for NO production in an insect hemolymph has been found in other insects. Mohamed et al. (2018) [38] showed the highest nitrite levels in the hemolymph of the flesh fly, *Sarcophaga argyrostoma*, injected with heat-killed gram-positive bacteria after 24 h, as well as Eleftherianos et al. (2014) [36] for *Drosophila* with different bacteria. In the fly *Chrysomya megacephala,* nitrate levels peaked 24 h after inoculation with yeast [32], and even in murine microglia cells, the highest nitrite levels indicative of NO synthesis were shown in response to LPS after 24 h [86]. In the mosquito, *Anopheles gambiae*, elevated NO synthesis [35] could at least partially be assigned to elevated NOS expression [33,44].

### 4.2. Responses to Different Immune Stimuli

Compared to inoculation with heat-killed gram-negative *E. coli* or gram-positive *S. suis,* we observed less NO production after inoculation with heat-killed gram-positive *S. aureus* regardless of the source of hemocytes. One possible explanation could be a very efficient clearing of *S. aureus* before the onset of NOS upregulation and/or activation. Immune mechanisms such as enhanced phagocytosis in *A. aegypti* or production of AMPs in *Drosophila* are discussed to be sufficient for clearing an *S. aureus* infection [87,88]. A second explanation would be the differential activation of immune pathways by the tested bacteria strains.

A common assumption is that gram-negative and gram-positive bacteria exhibit different peptidoglycans on their cell wall [89] and activate distinct immune pathways in insects. Gram-negative bacteria predominantly activate the IMD pathway, while gram-positive bacteria rather activate the Toll pathway [5]. However, at least in *Drosophila*, there is not always a strict link between bacteria classes and activated immune pathways. Some peptidoglycan recognition proteins can detect gram-negative bacteria but activate the Toll pathway [16], whereas other gram-positive bacteria activate the IMD pathway [8,14,17]. Furthermore, mixed responses are possible as well, exemplified by gram-positive *Bacillus subtilis* or *Bacillus thuringensis*, which can activate both pathways [8].

In addition, the heat inactivation of bacteria can have an influence on the evoked insect immune response. *G. mellonella* were found to be an eligible host model for both *S. suis* and *S. aureus* infection [90,91]. After injection of a viable *S. aureus*, *G. mellonella* showed an increase in hemocytes number and nodule formation, whereas injection of heat-killed bacteria resulted in an increase in antimicrobial peptides and enhanced ability to kill yeast cells [91].

For a full activation of the Toll pathway, additional factors other than peptidoglycan pattern recognition appear to be necessary for *Drosophila,* e.g., secreted bacterial proteases. An injection of heat-killed *Enterococcus faecalis* induced significantly less drosomycin than an injection of living bacteria [92]. A similar effect might apply for NO synthesis in response to *S. aureus* in *L. migratoria*. Both bacteria species utilize the critical regulatory system VraSR, which is involved in the building of the cell wall and promotes evading of the immune system in live bacteria [93,94]. Since heat-inactivated bacteria release cell contents such as DNA, cytoplasmic content, and cell wall components [95], other immune mechanisms might be activated. Although recognition of a specific pathogen is conserved during evolution [5], Buchon et al. [96] showed an alternative activation for the Toll pathway by gram-positive bacteria and fungi for *Drosophila* compared to other insect taxa [96]. Therefore, the elicited immune response seems to depend not only on the species of the bacteria but also the insect species.

Since both heat-inactivated gram-negative and gram-positive bacteria induced NOS activity in locust hemocytes in our experiments, we tested whether components of bacterial cell walls alone could elicit the same response. Using an amount of bacterial lipopolysaccharides (LPS) equivalent to or 10 times higher than the estimated LPS content of the tested *E. coli* dose, we saw neither citrulline immunoreactivity nor capsule formation (Figure 5). This is in line with the experiments of Goldsworthy et al. (2003) [29], who reported nodule formation in locusts only when injected with extremely high and deadly amounts of LPS. Likewise, physiological concentrations of the minimal component of bacterial proteoglycans, muramyl dipeptide (MDP), had no effect on NOS activity or capsule formation in our experiments. MDP is highly immunogenic in vertebrates, but the vertebrate MDP sensing receptor group of the NOD family appears to have no equivalent in invertebrates [89]. In addition, the hematopoietic tissue of the locusts is highly efficient in clearing substances from the hemolymph and nodules will only form when the phagocytic capacity is exceeded [24,70]. The effect of LPS could also be diminished by the physiological status of the locusts that had continuous access to food. In resting and fed locusts, the hemolymph contains high levels of high-density lipoprotein, which alleviates the toxic effect by binding to LPS [29,97,98,99].

Since soluble factors alone could not provoke NOS activation, we also tested bacteria-sized inert particles, PMMA beads, which were apparently phagocytosed but did not result in the induction of citrulline immunoreactivity in hemocytes or capsule formation, either. Furthermore, injection with RVFV viral particles did not result in NOS activation (Figure 6). However, we only confirmed the successful application of viral particles to the locusts (Figure 6A) but were not able to detect viral replication. Thus, locusts might not be susceptible to RVFV infection and do not promote viral replication. In comparison to bacteria, which are able to induce immune activation by inactivated pathogens [91], immune activation by replication-incompetent virus-like particles has not been described to the best of our knowledge. In conclusion, we were not able to detect NOS activation by the RVFV virus strain used, but this might be due to the lack of viral replication in our model system and may deserve further evaluation in other insect systems.

On the other hand, our semi-automatic assessment of microscopic images proved to be sensitive enough to show an effect for the sham-injected group when compared to the naive group in some cases. We cannot completely rule out contamination, but a wound reaction to the mechanical damage caused by the injection capillary is more likely. The integration of a wounding reaction and activation of cellular and humoral immune responses by damage associated molecular patterns in insects was already shown in *Drosophila* [100,101,102,103]. Regarding NO production, Faraldo et al. [32] noticed elevated nitrite levels in saline injected flies *C. megacephala* compared to uninjured flies but still lower levels compared to a yeast-injected group.

In our experiments, a consistent feature was not only the aggregation of hemocytes and capsules on the dorsal abdomen and along the heart but also frequent NO-producing hemocytes and capsules on the ventral abdomen and along or directly on the nervous system. This distribution pattern of capsules was also described by Goldsworthy et al. [29] after injection of high doses of LPS in *L. migratoria*. Another recent study surveyed over 60 insect species of different orders but observed very little pathogen-induced hemocyte aggregation on the ventral abdomen, for example in Orthoptera, which was based on the hemolymph circulation patterns [4]. However, in the latter study, dissection was performed at one to four hours after infection, which explains the discrepancy to our findings since capsule formation seems to be time dependent. Hemolymph circulation patterns along with position of the ostia can explain the predominant dorsal location of aggregates [4]. In addition, in locusts there is a hematopoietic tissue along both sides of the heart tube and its reticular cells show distinct macrophagic capacities [104,105]. Electron microcopy revealed large amounts of iron saccharate or latex beads were taken up shortly after injection in vacuoles of reticular cells [70], and Goldsworthy et al. [29] described nodules as producing a defined pattern reflecting the distribution of the reticular cells. In our experiments, similar capsule patterns occurred in abdomens with strong immune responses towards bacteria and phagocytotic patterns are pronounced in locusts injected with virus particles or fluorescent beads.

### 4.3. NO/cGMP Signaling between Immune and Nervous System

Although the immune reaction was more pronounced dorsally, we frequently found a strong accumulation of NO-producing hemocytes ventrally or directly attached to the nervous system in animals injected with *E. coli*, raising the possibility that NO released by theses hemocytes could influence the CNS. The canonical signal transduction pathway for NO involves activation of a soluble guanylyl cyclase (sGC), which synthesizes the second messenger, cGMP [106]. NO-induced cGMP synthesis can be visualized using an antibody against cGMP in preparations where hydrolysis of cGMP has been inhibited during NO stimulation [107]. In our preparations of locust embryos, exposed CNS ganglia in proximity to citrulline-immunoreactive hemocytes clearly displayed accumulation of cGMP in both cell bodies and neuronal processes including interganglionic axons projecting anteriorly towards the brain. There are several examples of an influence of the NO/cGMP signaling pathway on insect sensory processing and behavior. In locusts, photoreceptor cells are modulated by retrograde NO/cGMP signaling [108]. In crickets, the effects on long term memory and odor preference by the injection of NO donors and NO inhibitors were found [109]. Motoric functions such as grasshopper stridulation are under the inhibitory control of NO/cGMP [110]. In *Drosophila melanogaster*, visual working memory formation is regulated by the short time elevation of NO/cGMP signaling in single axonal branches of ellipsoid body neurons, where cGMP increases transmitter release via cGMP gated calcium channels [54]. Thus, altered neuronal activity is likely to be expected in neurons responding to NO released from immune-challenged hemocytes, with a potential influence on the insect’s behavior. It will be interesting to address the question as to whether NO/cGMP signaling can mediate the perception of an infection by the insect’s CNS. This would not only be of interest in locusts, as they are an economically important pest species, but also of great importance given the numerous vector species in insects and their transmitted pathogens dependent on the vector’s behavior.

### 4.4. Future Prospects

Since nitric oxide has been reported to be an essential component of the hemocyte-mediated immune response against bacteria in mosquitos [33] and is probably also involved in *Drosophila* immunity [36,43], it will be interesting to test compounds interfering with NO signaling on the survival of locusts infected with living bacteria and other pathogens. An important question is the nature of the most efficient immunogenic ligand to induce NOS activity in locust hemocytes. Whereas LPS appears to be a potent stimulator of hemocyte NO production in lepidopteran hemocytes [41], in *Drosophila*, different proteoglycans seem to be the most potent activators of the IMD pathway by gram-negative, and the Toll pathway for gram-positive bacteria [8]. Intriguingly, locust hemocyte NO synthesis differed between the investigated gram-positive bacteria, but not between gram-negative *E. coli* and gram-positive *S. suis*. Investigations on the distantly related, hemimetabolous locust could complement studies on holometabolous species and promote understanding of the general principles of insect immunity. The large size of locust hemocytes, and the recent discovery of a calcium sensor for locust cells [111] will enable us to study the role of calcium signaling in linking the immune stimulus to NO synthesis as a response. A further advantage of the locust is the large hemolymph volume and, thus, the large number of hemocytes that they share with other basal pterygotes. Advanced pterygotes have much lower hemolymph volumes as a consequence of optimizing for high-performance flight [112]. Large hemocyte numbers combined with pharmacological tools will enable studies on the cellular and biochemical levels in locusts that can complement studies on the genetic level in *Drosophila* or *Tribolium*.

## Data Availability

The data presented in this study are available in the supplementary information.

## Supplementary Materials

The following are available online at https://www.mdpi.com/article/10.3390/insects12100951/s1, Figure S1, Testing of preincubated anti-citrulline antibody with BSA-conjugated amino acids on dorsal abdomens of first instar locust injected with 5 × 10^5^ CFU heat-inactivated *E. coli* after 24 h; A control; B preincubated with BSA-conjugated arginine 100 mM (1:200) C; preincubated with BSA-conjugated citrulline 100 mM (1:200), asterisks indicate capsules. Figure S2, Average numbers of hemocytes gained from adult locusts of different treatment groups after hemocyte preparation. Naive: 329.389 ± 39.257 hemocytes per locust (n = 15, ±SEM), sham: 378.785 ± 38.210 hemocytes per locust (n = 16, ±SEM), *E. coli*: 403.802 ± 35.439 hemocytes per locust (n = 16, ±SEM), *S. suis*: 276.694 ±63.512 hemocytes per locust (n = 6, ±SEM), *S. aureus*: 348.333 ± 56.789 hemocytes per locust (n = 3, ±SEM), MDP: 401.750 ± 38.366 hemocytes per locust (n = 3, ±SEM), PMMA beads (2 µm): 368.834 ± 47.834 hemocytes per locust (n = 2, ±SEM), statistical test: Kruskal–Wallis test with Dunn’s multiple comparison test. Table S1, Number of preparations with citrulline-positive hemocytes, with capsules and total preparations of first instar locust abdomens: sham injected, 5 × 10^5^ CFU heat-inactivated *E. coli* injected, 5 mM D-NMMA and 5 × 10^5^ CFU heat-inactivated *E. coli* injected and 5 mM L-NMMA and 5 × 10^5^ CFU heat-inactivated *E. coli* injected. Table S2, Number of preparations with citrulline-positive hemocytes, with capsules and total preparations of first instar locust abdomens: naive locusts, LB injected, 5 × 10^5^ CFU heat-inactivated *E. coli* injected, 5 × 10^5^ CFU heat-inactivated *S. suis* injected and 5 × 10^5^ CFU heat-inactivated *S. aureus* injected. Table S3 Number of preparations with citrulline-positive hemocytes, with capsules and total preparations of first instar locust abdomens: (A) LPS injection, (B) MDP injection, (C) PMMA beads injection, (D) RVFV injection. Table S4, Numbers of adult locust primary hemocyte culture.
